# Establishment and analysis of artificial neural network diagnosis model for coagulation-related molecular subgroups in coronary artery disease

**DOI:** 10.3389/fgene.2024.1351774

**Published:** 2024-02-29

**Authors:** Biwei Zheng, Yujing Li, Guoliang Xiong

**Affiliations:** ^1^ Department of Cardiology, Dongguan Hospital of Integrated Chinese and Western Medicine Affiliated to Guangzhou University of Traditional Chinese Medicine, Dongguan, China; ^2^ Shenzhen Traditional Chinese Medicine Hospital, The Fourth Clinical Medical College of Guangzhou University of Chinese Medicine, Shenzhen, China; ^3^ Beijing University of Chinese Medicine Shenzhen Hospital (Longgang), Shenzhen, China

**Keywords:** coronary artery disease, coagulation, random forest, LASSO, artificial neural networks

## Abstract

**Background:** Coronary artery disease (CAD) is the most common type of cardiovascular disease and cause significant morbidity and mortality. Abnormal coagulation cascade is one of the high-risk factors in CAD patients, but the molecular mechanism of coagulation in CAD is still limited.

**Methods:** We clustered and categorized 352 CAD paitents based on the expression patterns of coagulation-related genes (CRGs), and then we explored the molecular and immunological variations across the subgroups to reveal the underlying biological characteristics of CAD patients. The feature genes between CRG-subgroups were further identified using a random forest model (RF) and least absolute shrinkage and selection operator (LASSO) regression, and an artificial neural network prediction model was constructed.

**Results:** CAD patients could be divided into the C1 and C2 CRG-subgroups, with the C1 subgroup highly enriched in immune-related signaling pathways. The differential expressed genes between the two CRG-subgroups (DE-CRGs) were primarily enriched in signaling pathways connected to signal transduction and energy metabolism. Subsequently, 10 feature DE-CRGs were identified by RF and LASSO. We constructed a novel artificial neural network model using these 10 genes and evaluated and validated its diagnostic performance on a public dataset.

**Conclusion:** Diverse molecular subgroups of CAD patients may each have a unique gene expression pattern. We may identify subgroups using a few feature genes, providing a theoretical basis for the precise treatment of CAD patients with different molecular subgroups.

## Background

Coronary artery disease (CAD) is a prevalent cardiac illness characterized by the narrowing or blockage of coronary arteries, which are the major vessels supplying blood to the heart. This restriction impedes the delivery of sufficient blood, oxygen, and nutrients to the heart muscle, leading to the accumulation of cholesterol deposits (plaque) and inflammation within the arterial walls (Libby et al., 2021). As a leading cause of death worldwide, CAD poses significant health risks and requires prompt intervention and management to mitigate its adverse effects. In 2019, CAD affected an estimated 197 million patients worldwide, resulting in 9.1 million deaths (16.1% of all deaths) (GBD, 2019 Demographics Collaborators, 2020; Roth et al., 2020). As with the majority of complicated disorders, A person’s risk of suffering CAD is influenced by the interplay of inherited and lifestyle variables (Khera and Kathiresan, 2017). The latest epidemiological studies have shown that risk factors for the development of CAD include smoking, hypertension, dyslipidemia, and lack of physical activity, while the prevalence of CAD is increasing in elderly, diabetic, and obese populations (Duggan et al., 2022).

Recent research has shed light on the significant roles of coagulation Factors II (prothrombin), V, VII, and X in CAD. Dysregulation levels of these factors are associated with an increased risk of CAD and adverse cardiovascular events. High neutrophil and basophil blood cell counts, linked to enhanced factor II plasma coagulation activity, may predict mortality in clinically stable CAD patients, indicating underlying prothrombotic mechanisms (Pizzolo et al., 2021). Additionally, the Factor V Leiden mutation poses a risk for premature coronary artery disease, while elevated levels of the coagulation factor VIIa-antithrombin complex are associated with an increased risk of ischemic stroke/systemic thromboembolism (Paszek et al., 2022; Agosti et al., 2023; Valeriani et al., 2023). In related experiments, high-dose statin therapy has shown effectiveness in reducing levels of coagulation factors VII, VIII, and XI, all linked to thrombosis (Stępień et al., 2023). Notably, the reduction in factor XI levels corresponds to a less prothrombotic fibrin clot phenotype, suggesting additional antithrombotic effects in CAD patients (Stępień et al., 2023). Specifically, Factor II promotes thrombus formation, Factor V facilitates fibrin formation, Factor VII initiates the coagulation cascade, and Factor X promotes clot formation, offering potential therapeutic targets for CAD management (Redondo et al., 1999). Furthermore, targeting fibrinogen and factor XI has been demonstrated to decrease the risk of venous thromboembolism and ischemic stroke, supported by Mendelian randomization analysis (Yuan et al., 2021). Additionally, inhibiting factors V, VII, and X may reduce the risk of ischemic stroke (Yuan et al., 2021). These findings underscore promising therapeutic targets for mitigating cardiovascular disease risk associated with the inhibition of clotting factors.

The coagulation system significantly impacts the development of atherothrombotic diseases such as atherosclerosis. Coagulation factors also contribute to plaque instability, inflammatory responses, and thrombotic events within arterial walls, exacerbating atherosclerosis progression and elevating the risk of cardiovascular events like myocardial infarction and stroke (Ajjan and Grant, 2006; Keihanian et al., 2018). Therefore, targeting coagulation system regulation presents a promising strategy for preventing and treating atherothrombotic diseases.

Hence, our study employs artificial neural networks to analyze coagulation-related gene expression patterns in CAD. This innovative approach deepens our comprehension of CAD pathogenesis by revealing intricate molecular signatures and interactions within the coagulation pathway. Through the identification of potential biomarkers and therapeutic targets, our research endeavors to propel personalized treatment strategies for CAD forward.

## Methods

### Publicly available cohort datasets and preprocessing

The “GEOquery” R package (Davis and Meltzer, 2007) was used to download data, and obtain the expression profiles of chip datasets GSE20681 (Beineke et al., 2012), GSE20680 (Elashoff et al., 2011), and GSE12288 (Sinnaeve et al., 2009). The chip probes corresponding to the platform were taken from the Gene Expression Omnibus (GEO) database. The “org.Hs.eg.db” R package was used to conversion to gene symbols. We combined the GSE20681 and GSE20680 datasets as the training group, and the GSE12288 dataset as the validation group. The “sva” R package was used to remove the batch effect of the two datasets due to differences in time, personnel, and processing methods (Leek et al., 2012). Principal component analysis (PCA) was used to assess the distribution of the two expression matrices.

Coagulation pathways were gathered from the Kyoto Encyclopedia of Genes and Genomes (KEGG) database (https://www.genome.jp/kegg/), including hsa04611 (platelet activation) and hsa04610 (complement and coagulation cascades) (Kanehisa and Goto, 2000). There are 209 genes in all determined to be coagulation-related genes (CRGs) in the two pathways.

### Consensus clustering analysis of CRG expression patterns

The k-means algorithm was used to cluster CAD samples with the same or similar expression levels of CRGs with 1000-times iteration for classification stability. We used the “ConsensusClusterPlus” R package to implement the algorithm for the optimal k-value (number of clusters) in the training cohort. PCA analysis was performed to reveal differences in the distribution of CRG-subgroups. We also used external datasets for validation.

### Pathway characteristics and immune landscape of CRG-subgroups

The heatmap was plotted to display changes in biological functions between CAD subgroups, gene set variation analysis (GSVA) was carried out using the “GSVA” R package (Hänzelmann et al., 2013) to evaluate normalized enrichment scores (NES) for pathway and functional annotations. The single-sample gene set enrichment analysis (ssGSEA) was used to quantity the degree of infiltration of 23 immune cell signatures in each coronary patient.

### Comparison and enrichment analysis of CRG-subgroups

The “limma” R program was used to obtain differentially expressed genes between the C1 and C2 CRG-subgroups (DE-CRGs). The “org.hs.eg.db” R package was applied to annotate gene symbols as Entrez IDs, and the “cluster Profiler” R program was used to perform Gene Ontology (GO) and KEGG pathway analyses on DE-CRGs.

### Identification and validation feature CRGs

First, the least absolute shrinkage and selection operator (LASSO) regression was used to filter feature DE-CRGs. The LASSO algorithm’s variable selection and shrinkage were performed using the “glmnet” R package (Friedman et al., 2010). For the training cohort, CRG-subgroup (C1/C2) of CAD was the response variable in the regression, while the independent variable in the regression was the normalized expression matrix of potential feature genes (DE-CRGs). The penalty parameter (λ) of the model was determined by ten-fold cross-validation following the minimum criterion. Then, a random forest (RF) model of DE-CRGs was created using the “randomForest” R package (Guidi et al., 2013), and dimension important values were extracted from the RF model using the approach of decreasing accuracy (Gini coefficient method). For further analysis, disease-specific genes with an importance value (“MeanDecreaseGini” index) higher than 2.0 were chosen. Finally, the feature DE-CRGs were obtained by intersecting the particular genes provided by the two approaches.

### Construction of CAD classification model by artificial neural network

Artificial neural network is a computing structure proposed based on the mechanism of biological neuron network, which is a kind of simulation, simplification and abstraction of biological neural network. Neurons (feature DE-CRGs) are the “nodes” of this network, the “processing units”. We constructed a topological network with layered connections. The neural network with layered structure can be separated into input layer (reception of external input information), hidden layer (exchange and transmission of internal information) and output layer (output of information processing results). Each layer is connected in sequence, and the signal is transmitted in one direction.

We used the training set dataset to establish the neural network disease classification prediction model, and another external data set (validation group) is selected for neural network model validation. The model of feature DE-CRGs was constructed using the “neuralnet” R package (Beck, 2018). Prior to training the neural network, normalization and min-max processing were performed on the two groups of data. In the neural network model, we set a hidden layer as a model parameter, and constructed a CAD classification model through the obtained gene weight information. In this model, the sum of the product of the weight score and the expression level of important genes is used as the disease classification score. The confusion matrix function was used to do the five-fold cross-validation and acquire the model accuracy results. The AUC classification performance verification results were calculated using the “pROC” software package. The accuracy, recall, precision, and F1 scores were evaluated to assess the validity and reliability of the model.

### Statistical analysis

All data analysis in this study was based on R software (version 4.2.1). Pearson and Spearman correlation analysis was used to test the correlation between two variables. Bayesian testing with Benjamini–Hochberg procedure were used for differential analysis to screen the genes with significant differences between the two groups. All tests were two-sided, and *p* < 0.05 was considered statistically significant.

## Results

### Characteristics of CRG-subgroups with coronary artery disease

**FIGURE 1:**
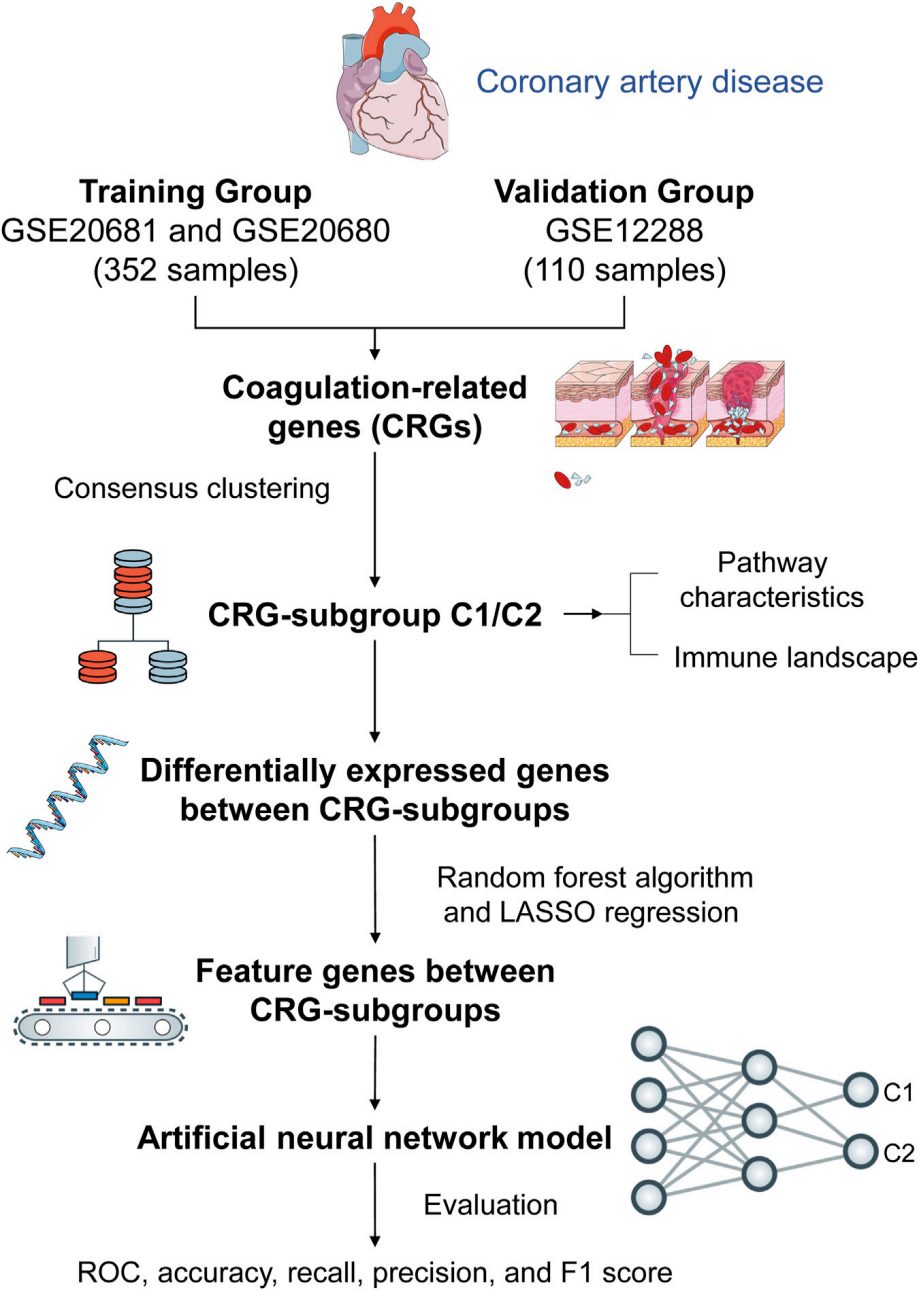
Workflow diagram.

**FIGURE 2:**
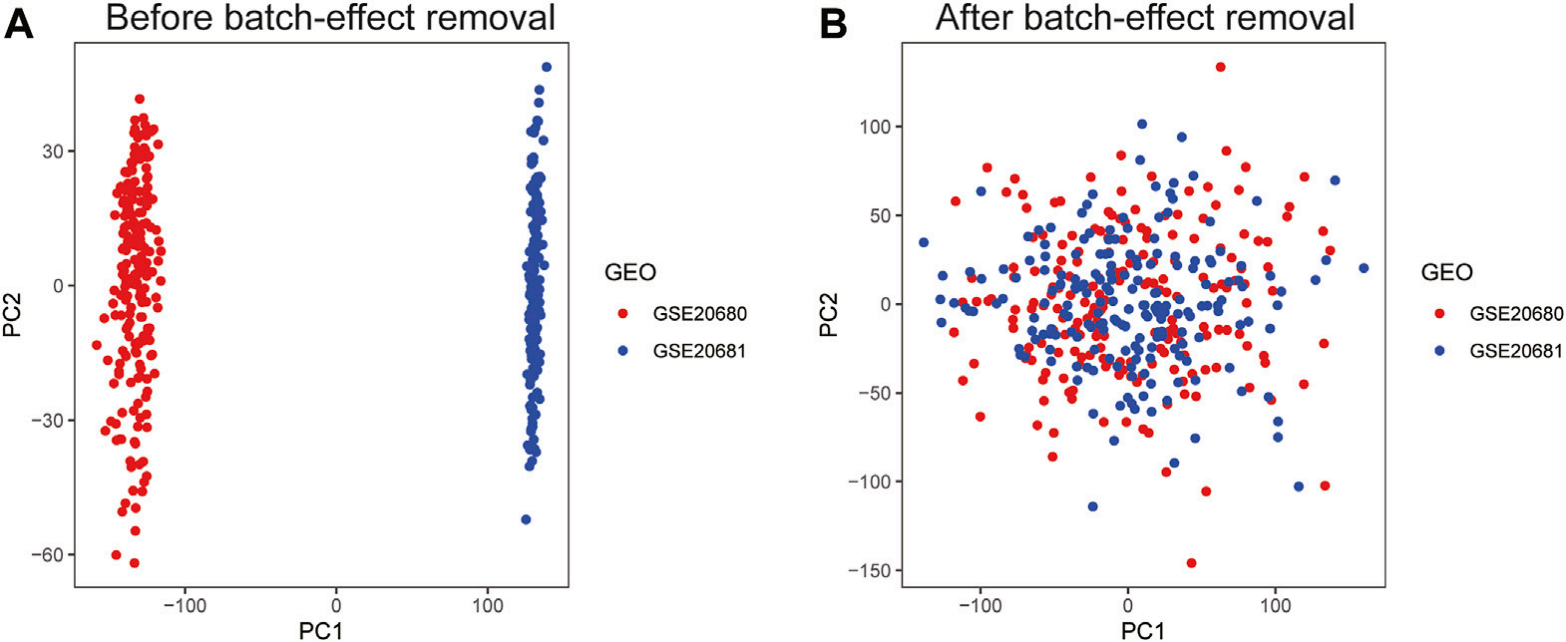
Principal component analysis (PCA) of the training group datasets. Visualization samples of the first two principal components before **(A)** and after **(B)** batch-effect removal.

The workflow diagram of the study is displayed in Figure 1. We obtained 242 CAD patients (excluding controls) from GSE20680 and GSE20681 datasets as the training group, and 110 CAD patients from GSE12288 as the validation group. We combined the data from the two datasets in order to eliminate the batch effect and get a consistent classification for the training group. Before removing batch effects, samples were clustered across datasets according to the first two principal components (PCs) of unnormalized expression values (Figure 2A). In contrast, the scatterplots of PCA analysis based on normalized expressions showed that the batch effect produced by different platforms was significantly eliminated (Figure 2B). The outcomes demonstrated that batch effect removal via cross-platform normalization is successful.

### Identification of CRG-subgroups in coronary artery disease

**FIGURE 3:**
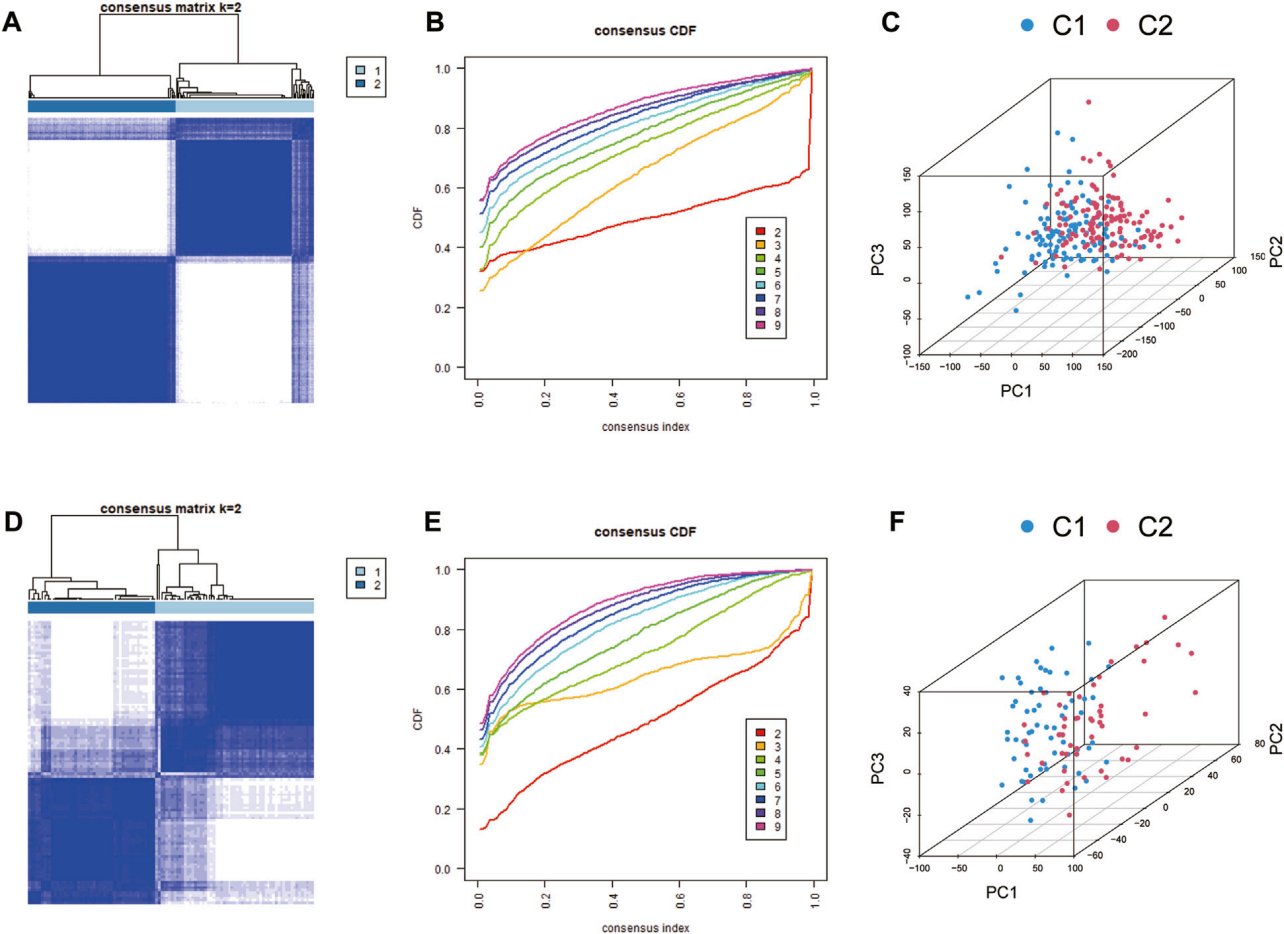
Identification of coagulation-related subgroups. **(A, B)** Consensus clustering matrix for k = 2 (optimal cluster number) of the training group. **(C)** PCA analysis of the training group. Cluster analysis **(D, E)** and PCA analysis **(F)** of the validation group.

Two distinct expression patterns, comprising 117 instances in the coagulation-related cluster C1 and 125 cases in cluster C2, were found by employing an unsupervised clustering approach to analyze the expression levels of CRGs from CAD patients in the training group (Figures 3A, B). In accordance with the PCA analysis, all patients could be roughly divided into two parts, which further confirmed two distinct subgroups (Figure 3C). Furthermore, we performed subgroup identification in the validation dataset. Similarly, the validation dataset can also be divided into two different coagulation subgroups (Figures 3D–F).

### Immune landscape of CRG-subgroups

**FIGURE 4:**
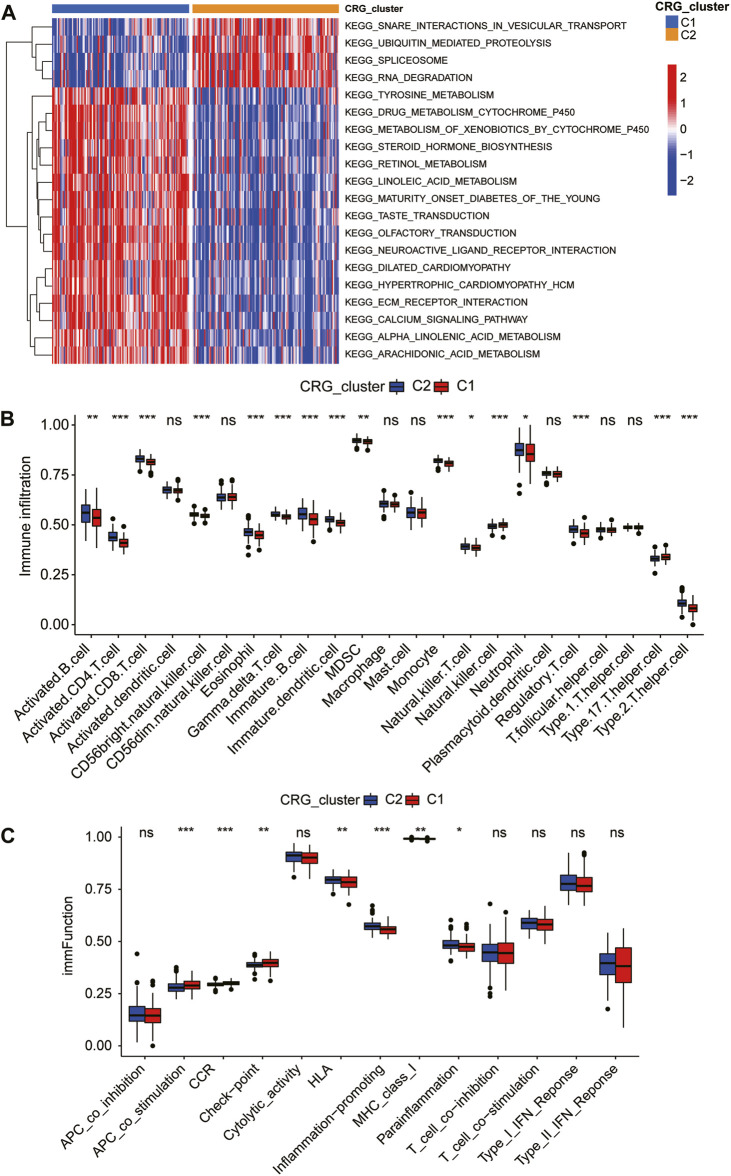
Immune landscape of coagulation subgroups. Gene set variation analysis (GSVA) **(A)** and differences in immune cell abundance **(B)** and immune indicators **(C)** between CRG-subgroups.

We used GSVA analysis to compare the regulatory pathway between the two coagulation subgroups in the training dataset, and we discovered that the two subgroups showed clear biological functional differences. The enrichment heatmap revealed that the signaling pathways involved in the metabolism of tyrosine, retinol, linoleic acid, and other biological compounds were significantly enriched in the C1 subgroup (Figure 4A). At the same time, we discovered that the C1 subgroup also had enriched calcium channels and ECM receptor interaction (Figure 4A). In 2022, it was proven that extracellular matrix proteins have a regulatory function on natural killer cells (Bunting et al., 2022). Calcium serves as both a signal and a nutrient in the regulation of numerous immunological responses linked to B cells and plasma cells (Newman and Tolar, 2021). Taking into account the relationships between CRG-subgroups and the immune system in CAD, we used the ssGSEA method and Wilcoxon test to analyze the abundance of immune cell infiltration of two CRG-subgroups based on the CRGs expression of the training group. The C1 subgroup is characterized by a higher degree of infiltration of natural killer cells and type 17 T helper cells; while the C2 subgroup is characterized by a higher degree of infiltration of immune cells such as activated B cells, activated CD4 T cells, activated CD8 T cells, eosinophil, and immature B cells (Figure 4B). We also calculated immune-related indicators using ssGSEA and found that the expression levels of antigen-presenting cell (APC) co-stimulation, check-point-related immune factors, and CCR gene family were higher in the C1 subgroup; the expression levels of HLA gene family, inflammation-promoting and parainflammation-related factors were higher in C2 subgroup (Figure 4C). This suggests that different coagulation subgroups of CAD have different immune microenvironments.

### Functional enrichment analysis of DE-CRGs

**FIGURE 5:**
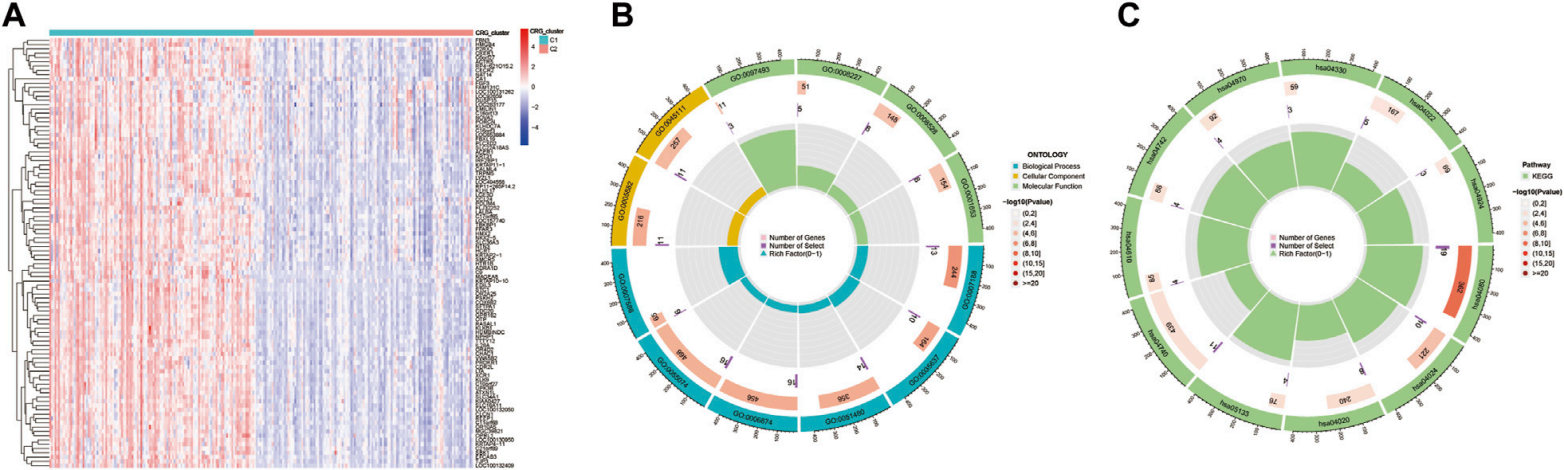
Functional analysis of DE-CRGs between two subgroups. Significance of difference analysis **(A)**, Gene Ontology (GO) enrichment analysis **(B)**, and Kyoto Encyclopedia of Genes and Genomes (KEGG) enrichment analysis **(C)** between C1 and C2 CRG-subgroups.

DE-CRGs between the C1 and C2 subgroups of the training set dataset were identified by Bayesian testing using the “limma” R package. We screened with |log2FC|>1 and adj. *p* < 0.05 as the threshold to identify 95 DE-CRGs related to the coagulation function of CAD, and the DE-CRGs are shown in the heatmap (Figure 5A; Supplementary Table S1).

GO enrichment analysis of 95 significant DE-CRGs was implemented using the “clusterProfiler” R package with the thresholds at *p*-values <0.01 and FDR values < 0.01. The outcomes of the GO analysis revealed that these DE-CRGs were mostly enriched in signal transduction-related biological functions such as G protein−coupled peptide receptor activity, serine−type endopeptidase activity, intermediate filament cytoskeleton, calcium ion homeostasis, and transmission of nerve impulse; they were also enriched in regulation of blood pressure, blood vessel diameter maintenance, positive regulation of vasoconstriction related to coagulation and vascular blood pressure regulation (Figure 5B; Supplementary Table S2). According to KEGG analysis, it was found that 95 DE-CRGs were enriched in neuroactive ligand-receptor interaction, cAMP signaling pathway, calcium signaling pathway, notch signaling pathway, complement and coagulation cascades and other signaling pathways (Figure 5C; Supplementary Table S3).

### Screening and validation of feature DE-CRGs

**FIGURE 6:**
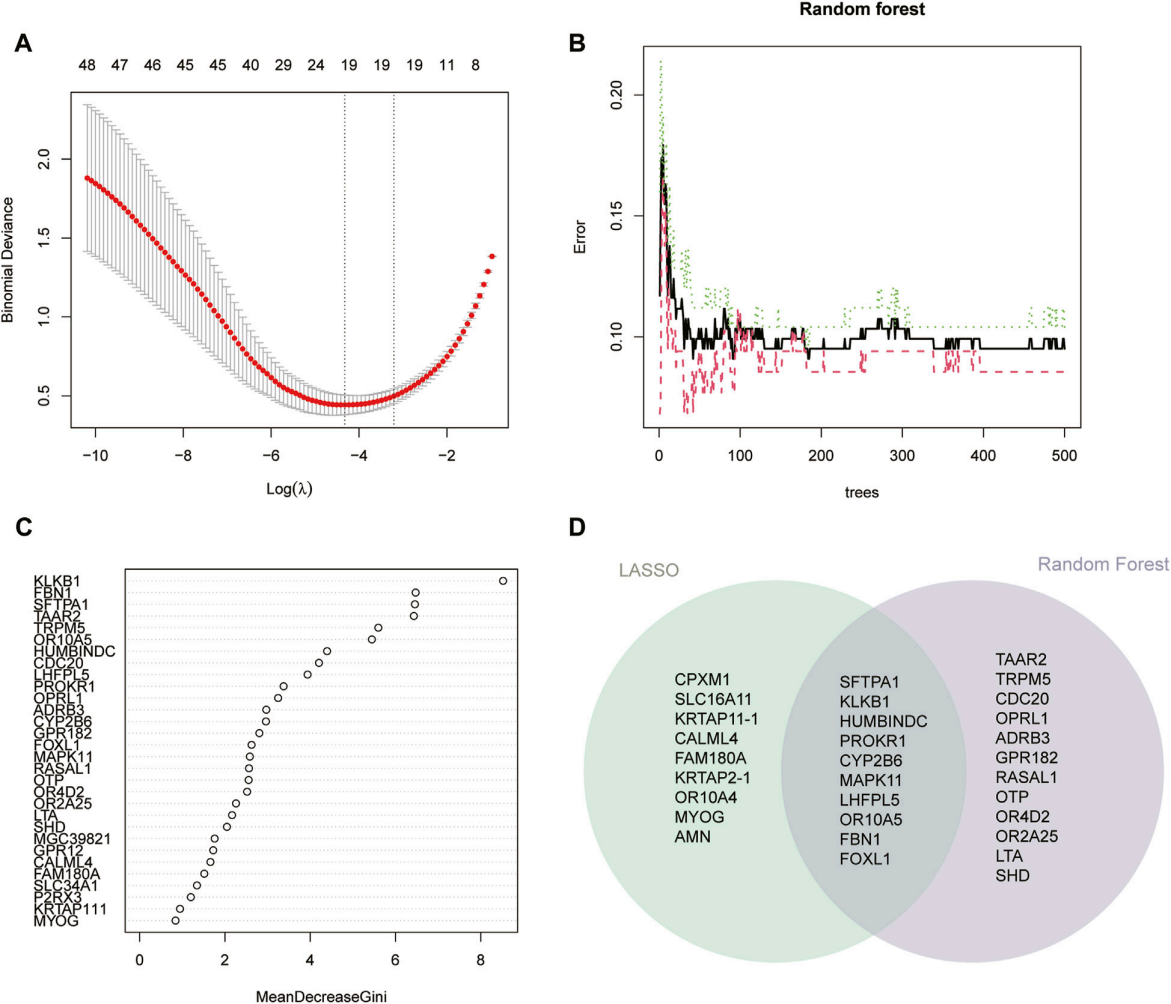
Identification feature DE-CRGs between two coagulation-related subgroups. **(A)** Cross-validation for selecting optimal parameter (λ) in LASSO regression. **(B)** Model error during building **(C)** and importance of top 30 genes in random forest model. **(D)** Intersection genes of 19 genes obtained by LASSO regression and 22 genes with “MeanDecreaseGini” index >2.0 in the random forest model.

First, LASSO regression analysis was performed on 95 DE-CRGs, and the cross-validation method was used for iterative analysis. The results showed that the model’s root means square error was lowest when there were 19 variables (Figure 6A). Then, we performed recurrent random forest classification on all possible numbers in 95 variables and calculated the average error rate of the model. Referring to the model error graph, it was found that when the number of classification trees is around 100, the error in the model tends to remain stable (Figure 6B). As the random forest model is being created, the Gini coefficient method was used to reduce the precision and mean square error, and the top 30 characteristic genes of variable importance were output, and the 22 characteristic genes whose “MeanDecreaseGini” index was greater than 2.0 were analyzed (Figure 6C). Finally, we intersected the genes acquired by the two methods to obtain 10 feature DE-CRGs (Figure 6D).

**FIGURE 7:**
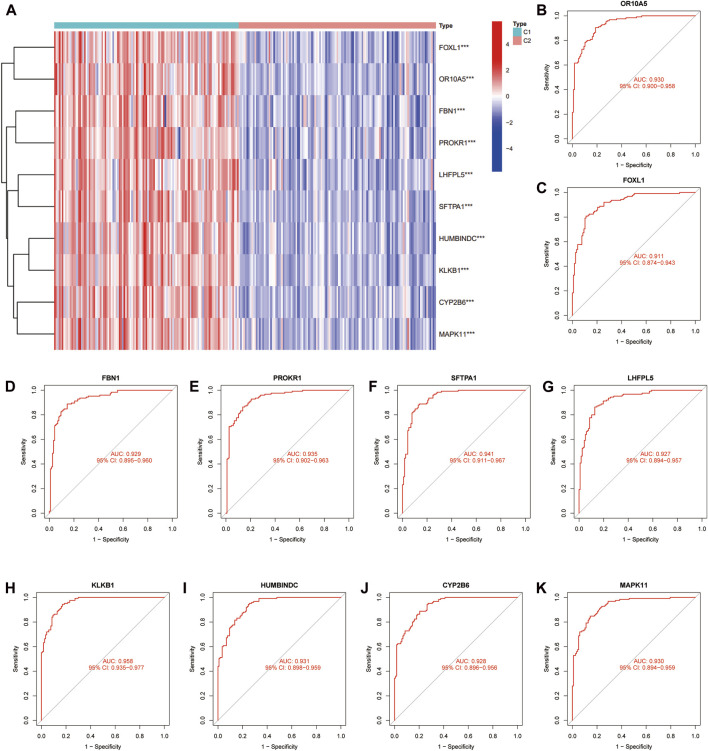
Evaluation and validation of the prediction performance of feature DE-CRGs. Unsupervised clustering **(A)** of 10 feature genes for C1 and C2 subgroups. ROC curves of *OR10A5* **(B)**, *FOXL1* **(C)**, *FBN1* **(D)**, *PROKR1* **(E)**, *SFTPA1* **(F)**, *LHFPL5* **(G)**, *KLKB1* **(H)**, *HUMBINDC* **(I)**, *CYP2B6* **(J)** and *MAPK11* **(K)** for predicting the coagulation-related subgroups.

We used unsupervised clustering to compare the expression levels of 10 feature DE-CRGs in CAD patients, and the findings revealed that C1 subgroup patients had high gene expression, whereas C2 subgroup patients had low gene expression. (Figure 7A). Subsequently, we constructed ROC curves for 10 feature DE-CRGs one by one to predict CAD coagulation-related subgroups, and found that the AUC values of 10 genes were all greater than 0.9 (Figures 7B–K). These results show that our feature DE-CRGs have an excellent ability to diagnose and predict molecular subgroups.

### Construction of artificial neural network model

**FIGURE 8:**
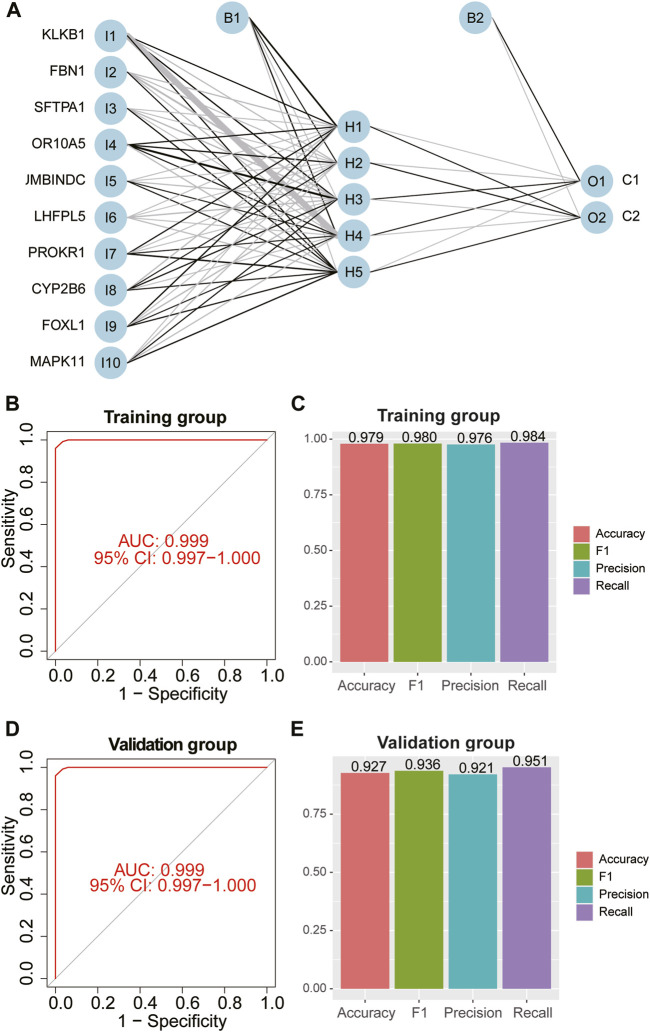
Construction and validation of the artificial neural network. Artificial neural network pattern plot **(A)** for predicting coagulation-related C1/C2 subgroups. ROC curves of training **(B)** and validation groups **(D)** for the model. The accuracy, F1-score, precision and recall of training **(C)** and validation groups **(E)** for the model.

We extracted a matrix of 10 feature DE-CRGs expression levels and CAD outcome variables (C1/C2) of 243 samples in the training group to establish a neural network prediction model (Figure 8A). 10 input layers, 5 hidden layers, and 2 output layers are set up for the artificial neural network. The area under the ROC curve (AUC) of the five-fold cross-validation results was 0.999 (Figure 8B). The accuracy, recall, precision, and F1 score of the training group were 0.979, 0.984, 0.976, and 0.980 (Figure 8C). Similarly, the classification effectiveness of the model scoring model created using gene expression and gene weights was confirmed using the validation group, and the AUC value of the ROC curve of the validation group also reached 0.999 (Figure 8D). The accuracy, recall, precision, and F1 score of the validation group were 0.927, 0.951, 0.921, and 0.936 (Figure 8E), which is confirmed that the artificial neural network model we established has excellent predictive robustness for the classification of C1/C2 CRG-subgroups in CAD patients.

**FIGURE 9:**
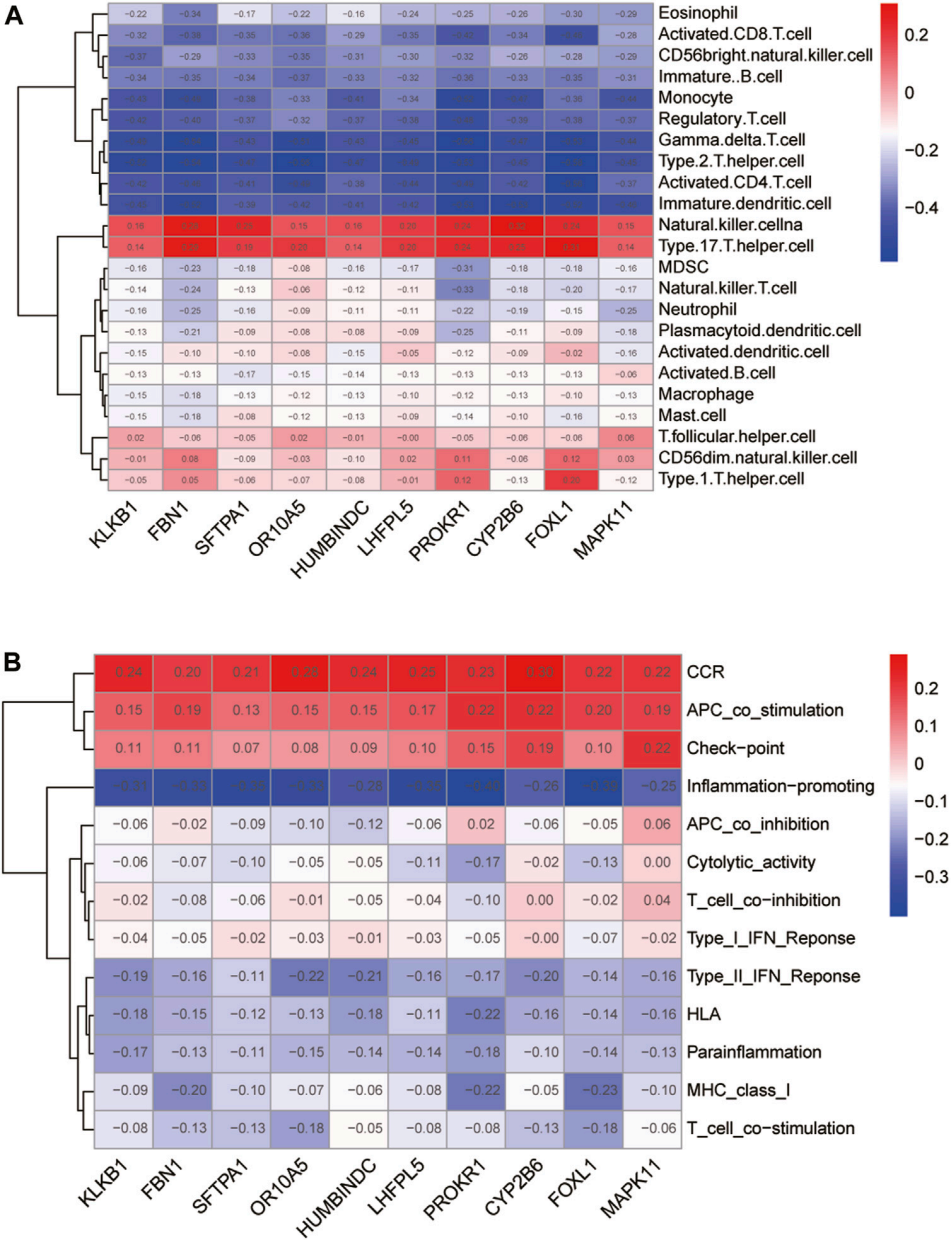
Immunological characterization of 10 feature DE-CRGs in artificial neural networks. Correlation of the 10 genes with immune cell abundance **(A)** and immune function **(B)**.

We performed Spearman correlation analysis on the gene expression levels of 10 feature DE-CRGs for constructing artificial neural networks and the relative abundance and immune function of immune cells. Among them, 10 genes were positively correlated with the contents of natural killer cells and type-17 T helper cells, and positively regulated the expression level of CCR family, the functions of APC co-stimulation and check-point; while 10 genes are negatively correlated with γδ T cells and type-2 T helper cells, and antagonize the immune function associated with inflammation-promoting and so on. All 10 genes were weakly correlated with immune-related indicators (|cor|<0.5, Figures 9A, B). Moreover, the correlation coefficients between the expression levels of different feature DE-CRGs and the content of a certain immune marker follow the nearly same pattern (a row of similar colors). We speculate that these 10 genes may collaboratively contribute to the immunological program of CAD patients.

## Discussion

In recent years, research on molecular subgroups and particular illness biomarkers has been intensively conducted due to the advancement of whole-genome sequencing technology and the demand for individualized treatment. For example, molecular characterization and typing of triple-negative breast cancer (Bareche et al., 2018; Zhao et al., 2020), colorectal cancer (Menter et al., 2019; Hu et al., 2021), lung adenocarcinoma (Wang et al., 2020), pancreatic ductal carcinoma (Topham et al., 2021), and other solid tumors based on tumor multi-omics datasets from the Cancer Genome Atlas (TCGA) database (Tomczak et al., 2015); molecular typing of non-neoplastic diseases are commonly used in immune-related diseases such as HIV (Patil et al., 2020; Amer et al., 2021). However, other diseases are limited by the lack of large-scale sequencing data, and molecular diagnosis and typing are still in the preliminary development. Therefore, we focused on the molecular characteristics of a special physiological activity (coagulation) in CAD patients using gene expression data from individuals in the GEO database. It is hoped that from the molecular level, as a supplement to traditional clinical diagnosis methods, it can improve the prediction accuracy of patient prognosis and diagnosis, and provide clinicians with improved decision-making tools.

In our study, an artificial neural network is introduced, and the model can accurately predict the molecular subgroups of CAD patients. An artificial neural network is an algorithm based on artificial intelligence and machine learning that consists of a densely linked network of computer processors that were inspired by biological nerve systems (Steimann, 2001). Backpropagation and Bayesian inference techniques are used in data mining and machine learning for artificial intelligence to handle gathered medical data (Salari et al., 2014). Artificial intelligence facilitates the clinical diagnostic and prognosis prediction processes by classifying and organizing medical knowledge and clinical data (Wong and Monaco, 1995; Jiang et al., 2017). The combined model of machine learning and artificial neural networks utilizing genetic polymorphisms in this study outperforms previous ANN models (Cheng et al., 2022) and other machine learning approaches (Peng et al., 2022) based solely on clinical features when diagnosing CAD patient subgroups. This enhanced diagnostic performance underscores the importance of integrating genetic information into predictive models for CAD. By leveraging genetic polymorphisms alongside ANN technology, this joint model offers improved accuracy in identifying CAD patients, thus potentially advancing personalized diagnostic approaches in clinical settings.

However, the limitations of the model are mainly reflected in the fact that the model may have overfitting problems when the training sample size is relatively small; at the same time, the initialization parameters of the neural network have a certain impact on the performance of the model, and the way to set the parameters is a non-deterministic polynomial problem. Therefore, one of the main directions that need to be explored in the next step is how to set the optimal initialization parameters of the model. This may involve combining regularization techniques with swarm intelligence optimization algorithms and ensemble methods to develop more reliable and generalizable models for genomic analysis. For example, the initial parameters of the model can be further optimized through the swarm intelligence optimization algorithm, such as the whale optimization algorithm (Brodzicki et al., 2021), Harris Hawks optimization algorithm (Qu et al., 2021), and wolf pack optimization algorithm (Chen et al., 2021), to obtain more accurate results. Additionally, efforts to improve data quality and increase sample sizes can help reduce the risk of overfitting and enhance the robustness of artificial neural network-based predictive models in clinical applications.

Among the feature genes for constructing artificial neural networks, *KLKB1* and *PROKR1* have been confirmed to be related to coagulation function and the angiogenesis process. *KLKB1* is usually synthesized in hepatocytes and secreted into the blood and is involved in the surface-dependent activation of blood coagulation, fibrinolysis, kinin production, and biological processes of inflammation, which can reflect the severity of liver injury (Che et al., 2021). The TBX20-PROK2-PROKR1 pathway may also be a target for the treatment of diseases associated with dysregulation of angiogenesis, benefit on patients with ischemic heart failure (Lichtenauer and Jung, 2018). Comparisons with similar studies might involve investigations into other receptor genes, such as *PROK2* (Lichtenauer and Jung, 2018), *EDN1* (Liang et al., 2018), and *NOS3* (Teralı and Ergören, 2019), which play roles in vascular function and inflammation regulation and may have similar implications in CAD.

*SFTPA1*, *FOXL1,* and *MAPK11* are tumor-characteristic molecular markers. *SFTPA1* variant carriers are at increased risk of inherited lung disease (Benusiglio et al., 2021), and this gene may be a viable prognostic biomarker since it is connected to immune cell infiltration and the effectiveness of immunotherapy in lung cancer (Yuan et al., 2022). It has been established that FOXL1 is intricately linked to the onset and progression of glioma (Chen et al., 2019), renal cancer (Yang et al., 2014), and pancreatic cancer (Zhang et al., 2013). *MAPK11* plays a role in a variety of female tumors (breast cancer (He et al., 2014), uterine endometrial cancer (Li et al., 2019), cervical cancer, ovarian cancer, and uterine carcinosarcoma), and its expression levels are significantly reduced (Katopodis et al., 2021). In addition, *CYP2B6* is the only gene encoding a functional enzyme in the human CYP2B subfamily (Desta et al., 2021), genetic variation in this gene locus affects the metabolism or bioactivation of clinically important drugs bupropion (Kirchheiner et al., 2003) and efavirenz (Haas et al., 2004; Desta et al., 2007). Pathogenic mutations in FBN1 are the cause of Marfan syndrome, a life-threatening autosomal dominant disorder of connective tissue (Wang et al., 2022). Lipoma HMGIC fusion partner-like 5 (*LHFPL5*) is an important molecule in the normal auditory system involved in mechanotransduction pathways in sensory hair cells of the ear (Yu et al., 2020).

It is noteworthy that these feature genes have been implicated in various biological processes relevant to immune function and CAD pathogenesis. For instance, *KLKB1* encodes for plasma kallikrein, which plays a role in the kinin-kallikrein system and has been associated with inflammation and thrombosis (Hayama et al., 2016). *PROKR1* has been linked to angiogenesis and vascular development, both of which are closely intertwined with immune response modulation (Goryszewska et al., 2020). Furthermore, *MAPK11* is involved in the MAPK signaling pathway, which regulates immune cell activation and cytokine production (Roche et al., 2020). By elucidating the interplay between these genes and immune cells, we gain insights into the complex immunological mechanisms underlying CAD development and progression. This understanding may inform the development of novel immunomodulatory therapies and precision medicine approaches targeting immune-inflammatory pathways in CAD.

The evolution of precision therapeutics in the context of disease genomics offers promising avenues for enhancing patient care in various medical conditions, including coronary artery disease (CAD). By leveraging extensive data analysis and molecular classification, precision medicine approaches aim to tailor treatments to individual patients based on their specific genetic makeup and disease characteristics.

In the study mentioned, the use of artificial neural networks represents a novel approach to identifying characteristic genes associated with CAD from large-scale genomic data. These genes can serve as diagnostic biomarkers, allowing for more accurate diagnosis and even risk prediction of CAD. The ability of artificial neural networks to analyze complex gene interactions enhances our understanding of the genetic mechanisms underlying CAD, thereby improving diagnostic accuracy, particularly in patients with diverse genetic backgrounds. However, despite the potential benefits, challenges remain. Large-scale, high-quality genomic data are essential for training and optimizing artificial neural network models, highlighting the need for continued investment in data collection and curation efforts. Additionally, further validation of the effectiveness and reliability of the identified feature genes in real-world clinical settings is necessary to ensure their utility in improving patient outcomes.

Future research directions could explore integrating multi-omics data, such as proteomics, to enhance artificial neural network model’s recognition capabilities further. Additionally, combining artificial neural networks with other advanced technologies like single-cell sequencing and gene editing may offer synergistic advantages in achieving more accurate and personalized diagnosis and treatment of CAD. In summary, precision therapeutics driven by advancements in disease genomics, coupled with innovative approaches like artificial neural networks, hold great promise for revolutionizing the diagnosis and treatment of CAD. Continued research efforts and technological advancements are crucial for overcoming existing challenges and realizing the full potential of precision medicine in cardiovascular healthcare.

## Conclusion

In summary, our study identified two distinct molecular subgroups in coronary artery disease (CAD) related to coagulation function through gene expression profiling of CAD patients. We further investigated the biological function and immunological characteristics of these subgroups, revealing differing immunological roles between them. Utilizing LASSO and RF, we screened feature genes associated with coagulation function and developed an artificial neural network model for subgroup classification. The model exhibited excellent prediction accuracy, providing a theoretical framework for precision medicine in CAD by identifying patients with different molecular subgroups and suggesting novel medication therapy targets. This research significantly advances precision medicine in CAD by aligning with personalized treatment strategies and offering new avenues for improving patient outcomes. Future directions involve validating these molecular subgroups in larger patient cohorts and exploring their implementation in clinical settings to realize the full potential of precision medicine in CAD management.

## Data Availability

The original contributions presented in the study are included in the article/Supplementary Material, further inquiries can be directed to the corresponding author.

## Abbreviations

CAD, Coronary artery disease; CRGs, Coagulation-related genes; DE-CRGs, Differential expressed genes between the two CRG-subgroups; GEO, Gene Expression Omnibus; GO, Gene Ontology; GSVA, Gene set variation analysis; KEGG, Kyoto Encyclopedia of Genes and Genomes; LASSO, Least absolute shrinkage and selection operator; PCA, Principal component analysis; RF, Random forest; ssGSEA, single-sample gene set enrichment analysis.
